# *In vitro* biotransformation assays using fish liver cells: Comparing rainbow trout and carp hepatocytes

**DOI:** 10.3389/ftox.2022.1021880

**Published:** 2022-09-23

**Authors:** Ina Bischof, Jon A. Arnot, Heinrich Jürling, Georg Knipschild, Christian Schlechtriem, Anna Schauerte, Helmut Segner

**Affiliations:** ^1^ Fraunhofer Institute for Molecular Biology and Applied Ecology, Schmallenberg, Germany; ^2^ Centre for Fish and Wildlife Health, University of Bern, Bern, Switzerland; ^3^ Department of Physical & Environmental Sciences, University of Toronto Scarborough, Toronto, ON, Canada; ^4^ ARC Arnot Research and Consulting, Toronto, ON, Canada

**Keywords:** rainbow trout, common carp, bioaccumulation, biotransformation, hepatocytes, *in vitro* assay

## Abstract

Biotransformation assays using primary hepatocytes from rainbow trout, *Oncorhynchus mykiss*, were validated as a reliable *in vitro* tool to predict *in vivo* bioconcentration factors (BCF) of chemicals in fish. Given the pronounced interspecies differences of chemical biotransformation, the present study aimed to compare biotransformation rate values and BCF predictions obtained with hepatocytes from the cold-water species, rainbow trout, to data obtained with hepatocytes of the warm-water species, common carp (*Cyprinus carpio*). In a first step, we adapted the protocol for the trout hepatocyte assay, including the cryopreservation method, to carp hepatocytes. The successful adaptation serves as proof of principle that the *in vitro* hepatocyte biotransformation assays can be technically transferred across fish species. In a second step, we compared the *in vitro* intrinsic clearance rates (CL_in vitro, int_) of two model xenobiotics, benzo[a]pyrene (BaP) and methoxychlor (MXC), in trout and carp hepatocytes. The *in vitro* data were used to predict *in vivo* biotransformation rate constants (k_B_) and BCFs, which were then compared to measured *in vivo* k_B_ and BCF values. The CL_in vitro, int_ values of BaP and MXC did not differ significantly between trout and carp hepatocytes, but the predicted BCF values were significantly higher in trout than in carp. In contrast, the measured *in vivo* BCF values did not differ significantly between the two species. A possible explanation of this discrepancy is that the existing *in vitro-in vivo* prediction models are parameterized only for trout but not for carp. Therefore, future research needs to develop species-specific extrapolation models.

## Introduction

For the aquatic environment, the most widely used parameter to estimate the bioaccumulation potential of a chemical is the bioconcentration factor (BCF) in fish. The standard approach for regulatory purposes to determine the BCF is the *in vivo* test according to the OECD Test Guideline (TG) 305 (OECD, 2012). The test is labor-intensive, costly, and requires a high number of animals (>100 per test). With the implementation of new chemical regulations worldwide (e.g., REACH), the need for bioaccumulation testing has increased extensively (Nichols et al., 2007; Gobas et al., 2009). The development of alternative testing strategies to reduce the need for *in vivo* BCF testing is therefore urgently needed (de Wolf et al., 2007; Lombardo et al., 2014).

A possible component of an alternative to the *in vivo* bioaccumulation assessment for fish is the use of *in silico* predictions of BCF values using Quantitative-Structure-Activity-Relationships (QSARs), mass balance models or physiologically based toxicokinetic (PBTK) models (Arnot and Gobas, 2003; Arnot et al., 2008; Nendza et al., 2018). A major uncertainty in the *in silico* predictions of *in vivo* BCF values, however, is the role of biotransformation which may substantially reduce the bioaccumulation potential of chemicals (Nichols et al., 2007). In absence of reliable biotransformation rate data, mass balance and PBTK models will overpredict BCFs for chemicals subject to biotransformation (Nichols et al., 2007; Nichols et al., 2009). QSARs for predicting biotransformation rate constants (and associated half-lives) in fish from chemical structure have been developed and validated (Arnot et al., 2009; Brown et al., 2012; Papa et al., 2014; Mansouri et al., 2018) following OECD QSAR guidance for use in regulatory applications (OECD, 2004, 2014). However, there is a need to expand the applicability domain of the QSARs by adding more diversity to the chemical training and testing sets, as well by evaluating inter-species differences in biotransformation rate constants. An approach to broaden the database on chemical biotransformation rates under avoidance of animal testing is the use of *in vitro* assays based on isolated liver cells or liver subcellular (post-mitochondrial) fractions (hepatic S9 fractions) of fish (Segner and Cravedi, 2001; de Wolf et al., 2007; Han et al., 2007; Nichols et al., 2007; Weisbrod et al., 2009; Kropf et al., 2020). These assays determine the depletion of a test compound over time (substrate depletion approach), and provide *in vitro* intrinsic clearance rates (CL_in vitro,_ _int_). *In vitro*-*in vivo* extrapolation (IVIVE) models can then be used to estimate *in vivo* intrinsic clearance rates (CL_in vivo,_ _int_), tissue clearance, i.e., hepatic clearance (CL_H_) and “whole body” biotransformation rate constants (k_B_) (Han et al., 2007; Nichols et al., 2013; Krause and Goss, 2018). For both the hepatocyte and the S9 assay, standard protocols and OECD Test Guidelines are available (Fay et al., 2015; Johanning et al., 2012; OECD, 2018a, b) so that they can be applied in regulatory testing.

The OECD TG 305 for *in vivo* BCF determination in fish accepts a broad variety of cold and warm water fish as test species, including rainbow trout (*Oncorhynchus mykiss*), common carp (*Cyprinus carpio*), fathead minnow (*Pimephales promelas*), medaka (*Oryzias latipes*), stickleback (*Gasterosteus aculeatus*), etc. (OECD, 2012). In contrast, the existing protocols for *in vitro* biotransformation assays are restricted to a single fish species, the rainbow trout. It is well documented that chemical biotransformation can differ strongly between fish species (Roberts et al., 2011; Bearr et al., 2012). It is thus critical to develop confidence in the inter-species scaling of biotransformation rates obtained from *in vitro* testing methods (Lin, 1998; Schultz and Hayton, 1999; Hutchinson et al., 2014). The question that needs to be answered is whether the *in vitro* assays for biotransformation assessment can follow a “one size fits all” approach, i.e., restrict the *in vitro* testing to a single species like rainbow trout, or whether they have to be expanded to *in vitro* preparations from diverse species.

The present study aims to compare biotransformation rate data obtained from *in vitro* assays with hepatocytes of a cold-water fish species, the rainbow trout, and a warm-water fish species, the common carp, *Cyprinus carpio*. More specifically, the questions addressed in this study are: i) Is it technically feasible to transfer the *in vitro* method established for rainbow trout hepatocytes to carp hepatocytes?, ii) can the resulting *in vitro* biotransformation rates be used to predict species-specific *in vivo* k_B_ and BCF values?, and iii) how do the predicted values compare to the *in vivo* values as determined by means of the OECD 305 Test Guideline for trout and carp?

Common carp has been selected as a second species since it is frequently used for BCF testing in Asian countries (Weisbrod et al., 2007), and it is the second most common fish species used for laboratory bioaccumulation experiments (Arnot and Gobas, 2006; Arnot and Quinn, 2015). As test compounds, we selected two xenobiotics which show rather different biotransformation rates in rainbow trout hepatocytes, namely benzo[a]pyrene (BaP), and methoxychlor (MXC). BaP shows relatively fast rates of biotransformation and MXC shows relatively slow rates (Fay et al., 2014b; Nichols et al., 2018). Both chemicals are hydrophobic compounds with relatively high log K_OW_ values (5.99 and 5.08, respectively) (Fay et al., 2014b). In addition to the *in vitro* assays, we performed *in vivo* studies according to OECD TG 305, since we were not able to find BCF data of comparable quality between rainbow trout and common carp. Finally, the present study also addressed the question whether the cryopreservation protocol as it has been established for rainbow trout hepatocytes (Mingoia et al., 2010), can be applied for carp hepatocytes as well.

## Materials and methods

All experiments of the present study were carried out in late summer in the same laboratory. The *in vitro* experiments were performed with the same batches of fish and identical procedures of sample preparation and analysis were used, minimizing potential technical sources of variability in biotransformation rates.

### Standards and reagents

Methoxychlor (MXC, 99.7% purity, used in the *in vivo* experiments) and benzo[a]pyrene (BaP, ≥ 96% purity) and Florisil^®^, 30–60 mesh, were obtained from Sigma Aldrich (Schnelldorf, Germany). ^14^C-MXC (3.96 MBq/mg, 98.4% radiochemical purity, used in the *in vitro* assays) was ordered from Quotient Bioresearch (Cardiff, UK). Deuterated methoxychlor (MXC-d_6,_ 99 atom % D) and benzo[a]pyrene (BaP-d_12_, 98.4 atom % D) were purchased from CDN Isotopes (Pointe-Claire, Quebec, Canada) and Labor Dr. Ehrenstorfer-Schäfers (Augsburg, Germany), respectively. Media, reagents, and material used for the *in vitro* hepatocyte experiments are specified in Bischof et al. (2016). All other reagents and solvents were of the purest grade available and purchased from commercial sources if not stated otherwise.

### Animals

Common carp (mirror carp, *Cyprinus carpio*) and rainbow trout (*Oncorhynchus mykiss*) used for liver cell isolation were obtained from Westerwälder Fischzucht Stähler (Hadamar-Niederzeuzheim, Germany) and as fertilized eggs from Sauerländer Forellenzucht Rameil (Lennestadt, Germany), respectively. The fish were raised to their experimental size at the Fraunhofer-IME-husbandry (see below). Rainbow trout used in this study were all female and sexually immature with an average gonadosomatic index (GSI) of <0.2 (Fay et al., 2014a) (Table 1). Common carp, in contrast, were from both sexes and their average GSI was 2.47–5.52, indicating sexual maturity. Further information on the animals used for cell isolation is given in Bischof et al. (2016). Rainbow trout (*Oncorhynchus mykiss*) and common carp (mirror carp, *Cyprinus carpio*) used in the *in vivo* bioconcentration tests were purchased as juveniles from Fischzucht Störk (Bad Saulgau, Germany) and Carus-ARF (Wageningen, Netherlands), respectively.

**TABLE 1:** Overview of the *in vitro* assays: Characteristics of hepatocyte donor fish and quality of cell isolation.

Chemical	Cells	n (run#)^a^ ^)^	Average body weight		Average GSI		Average cell yield^b^ ^)^		Average cell viability^c^ ^)**^	
			[G]	±SD	[x̅ ]	±SD	[10^6^/g liver]	±SD	[%]	±SD
Common carp										
MXC	fresh	6 (run 1–6)	592	85.3	5.52	3.62	58.5	9.45	77.4	4.62
BaP		6 (run 1–6)								
MXC	cryo	3 (cryo run 1–3)	563	175	2.47	1.43	75.6	16.5	87.0	7.46
BaP		3 (cryo run 1–3)								
Overall mean		9	582	111	4.38	3.25	63.4	13.2	80.6^*^	7.07
Rainbow trout										
MXC	fresh	5 (run 1–5)	231	22.0	0.198	0.029	66.8	13.0	96.1	1.79
BaP		4 (run 2–5)	237	18.5	0.204	0.029	71.7	7.96	95.9	1.95
MXC	cryo	4 (cryo run 1–4)	261	10.3	0.179	0.014	106	40.6	98.6	1.38
BaP		3 (cryo run 1,2,4)	261	12.5	0.181	0.016	123	37.9	98.1	1.48
Overall mean		9	244	23.2	0.189	0.024	81.4	31.2	97.1^*^	1.99

a N = number of individual *in vitro* experiments (*in vitro* runs) performed for the respective group. For different *in vitro* runs different batches of hepatocyte suspensions (non-pooled, derived from different cell isolations, each performed with one individual fish) were used. MXC, and BaP *in vitro* runs of the same species were performed in parallel using the same hepatocyte suspension.

b Average number of viable cells obtained per g liver ±standard deviation (SD). Significant differences were found neither between the overall means of cell yield calculated for trout and carp, nor between the different groups, i.e. average cell yields calculated for fresh and cryopreserved cells of carp and trout, respectively.

c Average % of viable cells in purified hepatocyte suspensions determined within 20 min after cell isolation ±standard deviation (SD). Average % cell viability of purified hepatocyte suspensions determined for cryopreserved cells within 20 min after thawing was 79.8 ± 9.59 and 75.7 ± 6.44 for carp and trout, respectively. For cryopreserved cells, average % recovery of viable cells after thawing (in relation to the number of cells initially cryopreserved) ± standard deviation (SD) was 25.8 ± 6.28 and 22.6 ± 7.07 for carp and trout, respectively.

^*^Indicates significant differences between species (*p* < 0.001; Mann-Whitney Rank-sum).

^**^Results were significantly different (*p* < 0.04; Kruskal–Wallis One Way Analysis of Variance on Ranks) between the groups trout cryo (98.6%) and carp fresh (77.4%), as well as between trout fresh (96.1%) and carp fresh (77.4%).

All fish were held at the Fraunhofer-IME-husbandry in charcoal-filtered and dechlorinated city water at a temperature of 11–16°C (trout) and 18–22°C (carp) and under a 16:8 light/dark cycle for several weeks or months until the study start. The fish were fed once daily with a commercially available extruded diet (Milkivit-type F-2P B40 for trout and Milkivit-type Pro Aqua K18 C3 for carp) at a rate of 1% of the animals’ average weight.

### Isolation and cryopreservation of hepatocytes

Isolation of hepatocytes from liver of rainbow trout was performed by a two-step collagenase perfusion procedure which is well established for rainbow trout (Nabb et al., 2006; Fay et al., 2015). Since carp liver (more precisely the hepatopancreas), in contrast to rainbow trout, does not possess a central portal vein, a different approach for liver cell isolation had to be used. In previous studies with common carp, an *in vitro* digestion method was used to isolate hepatocytes (Cowan-Ellsberry et al., 2008; Dyer et al., 2008). Here, we adopted the method of Segner et al. (1993) who used a two-step collagenase perfusion of carp liver via the *Arteria coeliaca* for cell isolation. With the perfusion method, the percentage of pancreatic cells in the final cell suspension is typically <5% (Vogt and Segner, 1997). Cryopreservation and thawing procedures applied for hepatocytes of both species were based on the standard protocols for rainbow trout (Fay et al., 2014a; Fay et al., 2014b). The viability of the cells was determined by means of the trypan blue exclusion assay and counting of stained and non-stained cells in a hemocytometer. The carp and trout primary hepatocyte suspensions used in the present study were aliquots of the *in vitro* preparations used in a previous study investigating the *in vitro* metabolite patterns of MXC (Bischof et al., 2016). A detailed description of the methods and materials used for the preparation of hepatocyte suspensions including the procedures for cell isolation, cryopreservation, and thawing, as well as cell counting, diluting to the target cell concentration, and heat-inactivating can therefore be taken from Bischof et al. (2016) and from the cited publications.

### *In vitro* hepatocyte clearance assays

The *in vitro* hepatocyte clearance experiments were performed in a substrate depletion approach according to the methods previously published by Fay et al. (2014b). These methods are in accordance with the procedures provided in the OECD TG 319A on the performance of *in vitro* hepatocyte clearance assays. In the present study, several *in vitro* runs with MXC and BaP using suspensions of either freshly isolated or cryopreserved hepatocytes from common carp and rainbow trout were carried out. For the different *in vitro* runs non-pooled hepatocytes were used, i.e. hepatocytes from different individual fish. For each run, triplicates of live cells (3 × 1 ml at nominal 2 × 10^6^ cells/mL) and heat-inactivated cells as controls were dosed with the test chemical dissolved in acetonitrile (ACN) at the targeted concentration (2 µM for MXC and 0.5 µM for BaP) and incubated for 4 h at 15 °C (trout) or 20 °C (carp). MXC and BaP reactions were run side by side in the same *in vitro* run using the same hepatocyte suspensions. The reactions were stopped after 5, 15, 20, 30, 60, 120, 180, and 240 min by pipetting aliquots (100 µL) of the cell suspensions into centrifuge tubes containing 400 µl methylene chloride (DCM) spiked with MXC-d_6_ at 0.4 µM (MXC reactions) or ice-cold ACN (BaP reactions). Samples were vortexed and centrifuged at 20,000 *g* for 10 min. The organic phase was then transferred to analytical vials and subjected to chemical analyses.

Table 1 provides an overview of the *in vitro* hepatocyte clearance experiments conducted in this study. In addition, characteristics of the donor fish such as the average body weights and GSI values as a measure of sexual maturity, and relevant information about the success of hepatocyte isolation such as the average cell yields and viabilities are specified for the different *in vitro* runs.

### *In vivo* bioconcentration tests

*In vivo* BCF values for MXC and BaP in carp and trout were determined in bioconcentration tests with both species in accordance to OECD TG 305 (OECD, 2012). The studies were approved by the State Agency for Nature, Environment and Consumer Protection (LANUV) North Rhine-Westphalia. The fish were exposed to a single concentration of both test items in a flow-through system for 35 days. No control groups were used. Dosing was performed by a solid-phase desorption system which was integrated in the fresh water supply allowing for a constant delivery of test concentrations to the fish chambers. The procedure of column generated concentrations was described in detail by Schlechtriem et al. (2017). For the present study, two packed columns containing each 500 g Florisil^®^ loaded with 200 mg of either MXC or BaP were used for each fish chamber. The uptake (exposure) phase of the study was followed by a 35 days depuration phase in uncontaminated water. The flow-through rate was maintained at 38.6 L/h for carp and 30.1 L/h for trout throughout the test. On days 0, 7, 14, 21, 28, 31, and 35 of the uptake phase and on days 4, 10, 21, and 35 of the depuration phase each time four fish from each test species were sampled for chemical analysis. Additional 2-4 fish were sampled each, at the start and at the end of the exposure period, as well as at the end of the depuration period for lipid analysis. The total lipid content of the fish was measured individually by gravimetric analysis according to Smedes (1999).

Water samples (400 ml) were taken from the test chambers at least three times per week. Preparation for chemical analysis was performed by liquid-liquid extraction using n-hexane (10 ml) containing deuterated MXC (35 ng). The purified organic phase was subdivided into two equal parts and evaporated to dryness under a stream of nitrogen. One part of the residue was dissolved in 200 µL DCM for MXC analysis. The other part was mixed with 1 ml ACN: water (8:2, v/v) and subjected to BaP analysis. Whole fish samples were prepared for chemical analysis individually. After grinding the tissue with the addition of dry ice in a Thermomix™ 31 (Vorwerk), the finely powdered homogenate was lyophilized using an AdVantage freeze dryer (Ismatec). Aliquots of the dry samples (0.5 g) were spiked with deuterated internal standards (5 ng MXC-d_6_ and 0.2 ng BaP-d_12_) and extracted three times with acetone: DCM (1:1, v/v) (10 ml). The pooled extracts were then extracted with ultra-pure water (45 ml) and mixed with sodium chloride (5 g) to remove acetone and matrix. The organic phase was concentrated (0.5 ml) and dissolved in cyclohexane: DCM (1:1, v/v) (4.2 ml). This was subjected to gel permeation chromatography (GPC) (GILSON system equipped with pump 307, syringe pump 402, GX-271 liquid handler, 508 interface module, and Trilution^®^ LC software; column i. d. 1.5 cm, material Bio-Beads S-X3, 200–400 mesh, Bio-Rad, flow rate 2 ml/min). Eluates containing MXC (26–31 min retention time) and BaP (32–38 min retention time) were evaporated to dryness and dissolved in hexane (1 ml) and toluol (50 µL), respectively. The collected MXC extracts were further concentrated by solid-phase extraction (SPE, Sep-Pak^®^ Vac 12cc, 2 g, silica cartridges, Waters) using DCM: n-hexane (50:50, v/v) as eluent. Eluent and hexane were used to equilibrate and condition the column before sample loading and subsequent washing with DCM: n-hexane (30:70, v/v). The eluate containing MXC was dried down and reconstituted in toluol (0.5 ml).

### Chemical analysis

MXC in the organic phase of samples from the *in vitro* incubations and in DCM extracts of water samples from the *in vivo* bioconcentration tests were analyzed by gas chromatography-mass spectrometry (GC-MS). The GC-MS system consisted of a HP5890 series II GC coupled to a HP5972 mass sensitive detector (Hewlett-Packard). Separations were performed on 5% phenyl polysilphenylene-siloxane capillary columns (BPX-5, 30 m length x 0.25 mm i. d, 0.25 µm d_f_, SGE). Analysis of BaP in ACN extracts of samples from the *in vitro* incubations and in ACN/water extracts of water samples from the *in vivo* bioconcentration tests was carried out by liquid chromatography (LC)-fluorescence detection on a HPLC Summit system equipped with an Ultimate 3,000 pump and a RF 2000 fluorescence detector (Dionex). Separations were performed on a Luna C18 100A column (Phenomenex, 250 mm × 4.6 mm, 5 μm particle size). Toluol extracts of fish samples from the *in vivo* bioconcentration studies were analyzed for MXC and BaP by GC-MS using a Varian Bruker 450-GC coupled to Varian Bruker 320-MS. Separations were performed on 1,4-bis(dimethylsiloxy)phenylene dimethyl polysiloxane capillary columns (Rxi®-5Sil MS, 20 m length x 0.18 mm i. d, 0.18 µm d_f_, Restek). A detailed description of the instrument conditions used for GC-MS and LC-fluorescence analysis is provided in appendices (Supplementary Appendix A.1).

### *In vitro* intrinsic clearance calculation

Measured values of parent chemical concentration were log_10_-transformed and plotted against time. A linear regression was performed on the substrate depletion data to estimate a first order elimination rate constant (k; equal to −2.3 × slope) with units of inverse time (1/h). *In vitro* intrinsic clearance values (CLin vitro, int, mL/h/10^6^ cells) were then obtained by dividing k by 2 × 10^6^ cells/mL, the nominal concentration of viable hepatocytes in the *in vitro* experiment (Fay et al., 2014b).

### Extrapolation of k_B_ and prediction of BCF

The IVIVE-BCF model of Nichols et al. (2013) for rainbow trout was applied. In contrast to previous approaches, this model predicts the BCF for a reference rainbow trout (10 g fish held at 15 °C and with a whole body lipid content of 5%), accounting for the scenario in most of the *in vivo* BCF tests. With algorithms that adjust for user-specified changes, the new model provides flexibility with respect to settings of fish holding temperature, lipid content, and body mass, as well as several other extrapolation factors. The model was used in a study by Fay et al. (2014b) investigating the intra- and interlaboratory reliability of the trout hepatocyte assay, and was shown to deliver improved BCF estimates when *in vitro* biotransformation rate data were incorporated. A drawback is that the Nichols et al. (2013) model is not parameterized for carp. We introduced some modifications to adopt the trout model for carp. Instead of the specific fractional liver weight of 0.015 g/kg for trout, for carp we used 0.0174 g/kg as assumed in the carp extrapolation model of Cowan-Ellsberry (Cowan-Ellsberry et al., 2008). In the same way, the equation used for estimating fish growth (k_G_) was adopted for carp. For trout, fish growth was calculated according to the algorithm given in the Nichols model. Furthermore, the fish holding temperature was changed from 15 to 21°C for carp and to 12°C for trout, representing the actual conditions in the *in vivo* tests carried out as part of this study. The settings for lipid content (9.08% for trout and 9.57% for carp) and body mass (18.5 g for trout and 44.6 g for carp) for the modeled fish were adapted according to the values measured in the experimental fish. A hepatocellularity value of 510 × 10^6^ hepatocytes/g liver was used for both species to extrapolate CL_in vitro, int_ to the whole liver. This hepatocellularity value has been determined empirically for rainbow trout liver (Fay et al., 2014a) and was used in a recent application of the Nichols prediction model (Fay et al., 2014b). However, it should be kept in mind that hepatocellularity can strongly vary with the physiological conditions, as the size of fish hepatocytes depends on the amount of stored energy reserves (Segner and Braunbeck, 1990). An important parameter in the extrapolation model is the unitless *f*_u_ parameter which accounts for the difference of chemical binding to proteins *in vitro* and *in vivo*. While some studies used a default *f*_*u*_ of 1, in the present study we used a *f*_*u*_ estimated by QSAR. Due to the lack of species-specific information, the default algorithms for calculating *f*_u_ as given by the Nichols model were also used for carp. All other equations and parameters were used as suggested by Nichols et al. (2013).

### *In vivo* bioconcentration test derived k_B_ and BCF

In addition to the *in vitro* biotransformation assay data, also the *in vivo* BCF test data were used to determine k_B_ values for carp and trout. The BCF values were calculated as steady-state bioconcentration factor (BCF_SS_) according to OECD TG 305 by dividing the mean of measured concentrations in fish (C_f_) at steady-state through the time-weighted average (TWA) concentration in water during the uptake phase. The decrease of test substance during the depuration phase was calculated by fitting an exponential function (C_f_ = C_f_,_0_ × e^(−kT×t)^), where C_f_,_0_ is the concentration at the start of the depuration period to the concentrations measured in fish, resulting in the depuration rate constant (k_T_) (OECD, 2012). Due to unexpectedly rapid uptake kinetics for both test substances, it was not possible to calculate the uptake rate constant (k_1_) based on the measured concentrations in fish during the uptake phase. For *in vivo* k_B_ estimation, the k_1_ values were therefore estimated using the kinetic mass balance modelling method of Arnot et al. (2008). The k_B_ values were then calculated as k_B_ = k_T_−(k_2_+k_E_ + k_G_), where k_2_, k_E_, and k_G_ are rate constants (1/d) representing chemical elimination from the organism via the respiratory surface, fecal egestion, and growth dilution. The model was parameterized maximizing the available measured physiological (e.g. whole body wet weight, lipid content) and exposure (e.g. temperature, dissolved oxygen concentration) data from the laboratory BCF study (Arnot et al., 2008). Details of the evaluation of the *in vivo* data for BCF calculation and of the method used for modeling the BCF parameters are given in appendices (Supplementary Appendix A.2). To distinguish the k_B_ and BCF values predicted based on the *in vitro* biotransformation data, in the following the k_B_ and BCF values derived from measured *in vivo* data are referred to as experimentally determined or *in vivo* k_B_ (k_B,__*in vivo*_ _estimate_) values and experimentally determined or measured BCF (BCF, _*measured*_) values.

### Statistics

Differences between groups were analyzed as appropriate by Student’s t-test, Mann-Whitney Rank-sum test, One-way ANOVA, or Kruskal–Wallis One-way ANOVA on ranks followed by an all pairwise multiple comparison procedure using SigmaStat^®^ 3.5 Software (Systat).

## Results

### Yield and viability of hepatocytes

Cell isolations performed by the two-step collagenase perfusion technique were successful for both species. They resulted in comparable average cell yields of 63.4 ± 13.2 and 81.4 ± 31.2 × 10^6^ viable cells per g liver for carp and trout, respectively (Table 1). The average percent cell viability of freshly isolated hepatocyte suspensions was significantly greater for trout compared to carp, with 97.1 ± 1.99% and 80.6 ± 7.07%, respectively. In contrast, the cell viabilities for suspensions of cryopreserved hepatocytes after thawing were comparable between carp and trout, with on average 79.8 ± 9.59 and 75.7 ± 6.44%, respectively. In addition, for both species, a comparable percentage of viable cells could be recovered after thawing in relation to the number of cells initially cryopreserved with 25.8 ± 6.28% and 22.6 ± 7.07% for carp and trout, respectively.

### *In vitro* intrinsic clearance

**FIGURE 1:**
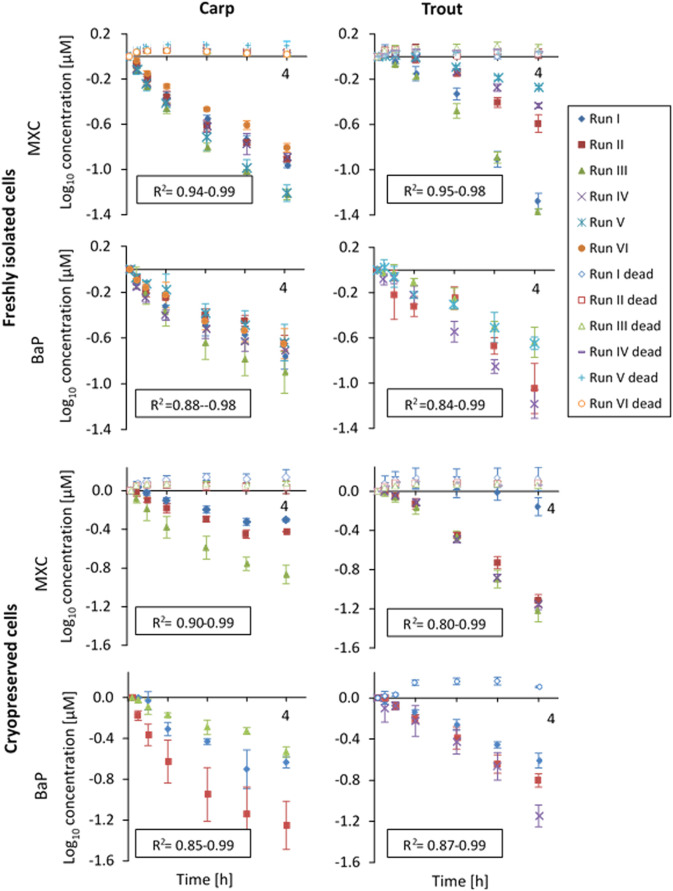
*In vitro* substrate depletion curves (measured concentrations were log_10_-transformed and normalized to the first sampling time point) from biotransformation rate assays with freshly isolated or cryopreserved common carp and rainbow trout hepatocytes. The cells were incubated over a 4-h-period (*x*-axis). Data points represent average substrate concentrations for each experimental run over the 4 h incubation period. Error bars indicate the standard deviation between triplicates. R^2^-values of the linear regressions are given for live replicates as a measure of goodness of fit. “Dead” indicates control values obtained with heat-inactivated hepatocytes.

Figure 1 shows the substrate depletion curves for MXC and BaP in carp and trout hepatocytes. The values were normalized to the first sampling time point for better comparability between the experiments. For both species, significant depletion of MXC and BaP was observed in freshly isolated and in cryopreserved hepatocytes. The depletion rates were comparable between freshly isolated and cryopreserved cells and log-linear, confirming first order kinetics. In cryopreserved carp hepatocytes the biotransformation activity slowed down toward the end of the incubation period. Therefore, for these experiments CL_in vitro, int_ rates were determined excluding data from the 4 h sampling time point using only the linear portion of the curve. Within the heat-inactivated controls (“dead” cells), the loss of parent chemical in no case exceeded 5% of the clearance rate of the live cells. The standard deviation between the three technical replicates of an *in vitro* run (intra-assay variability, error bars in Figure 1), generally tended to be higher for BaP compared to MXC. This finding might be attributed largely to the absence of an internal standard for BaP, which could compensate for the variability of the measured concentrations resulting from sample preparation and analysis.

**FIGURE 2:**
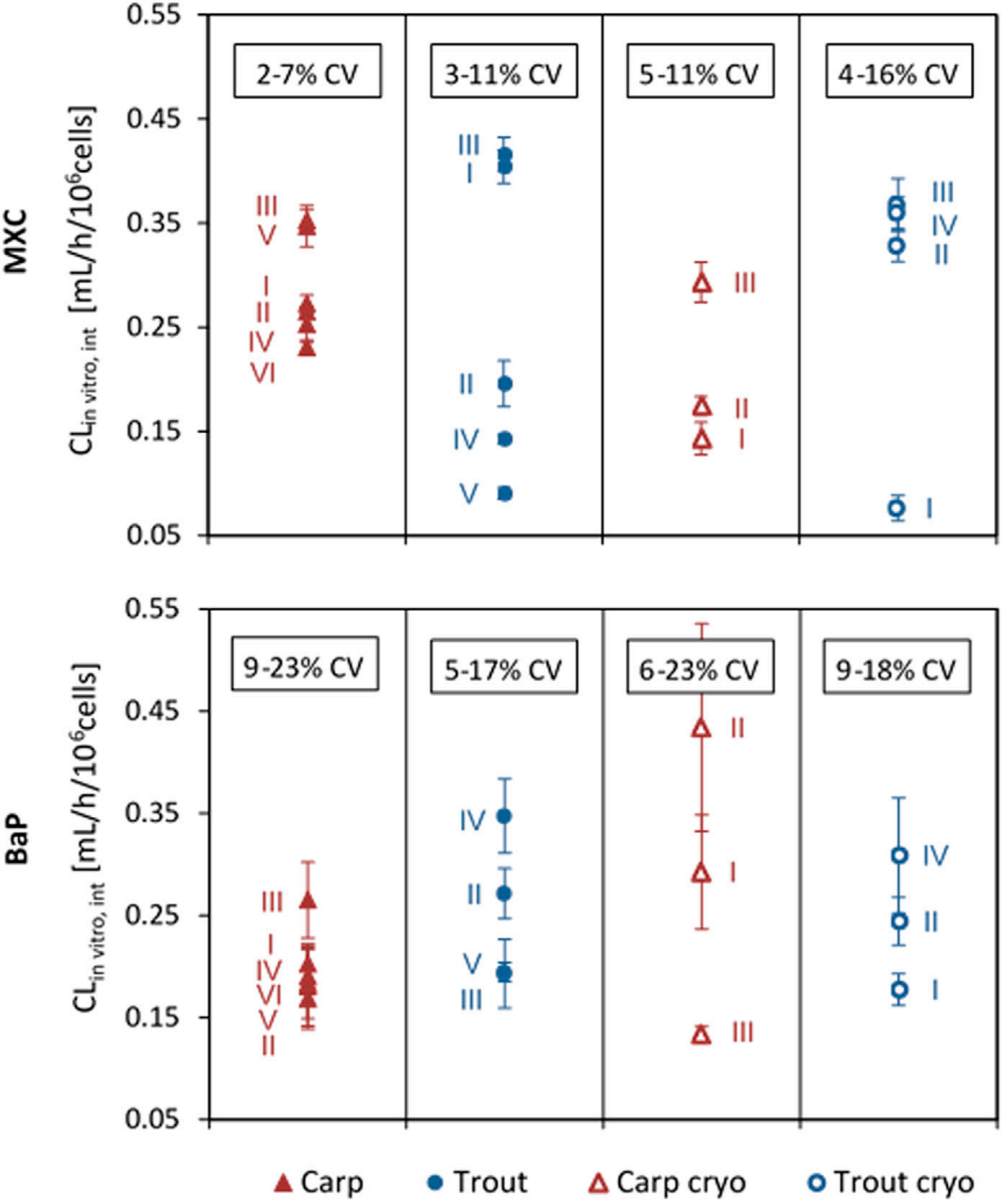
*In vitro* intrinsic clearance (CL_in vitro, int_) of methoxychlor (MXC) and benzo[a]pyrene (BaP) in common carp and rainbow trout hepatocytes. Values are normalized to 10^6^ cells, using the nominal test concentration of 2 × 10^6^ cells/mL. Values represent the average CL_in vitro, int_ from technical triplicates for each run. Error bars indicate the standard deviation (SD) between triplicates. The range of total coefficient of variation (%CV) values obtained for the triplicates in the different groups is provided in the boxes.

Average CL_in vitro, int_ values of MXC and BaP calculated for the independent biological replicates of the *in vitro* runs are plotted in Figure 2. The values obtained for the same fish species and the same chemical varied up to a factor of 5. This variability may confound existing inter-species differences.

Overall mean CL_in vitro, int_ rates determined for MXC and BaP in carp and trout hepatocytes are summarized in Table 2. Mean measured CL_in vitro, int_ values of hepatocytes of both species were in the range of 0.198 and 0.287 ml/h/10^6^ cells. Significant differences of MXC and BaP clearance rates were found neither between the two fish species nor between freshly isolated and cryopreserved cells. Also the mean CL_in vitro, int_ values of the two test chemicals did not differ significantly, except of freshly isolated hepatocytes of carp (Table 2). The *in vitro* intrinsic clearance rates differed between carp and trout hepatocytes with respect to the observed variabilities of the mean CL_in vitro, int_ rates: The inter-assay variability (variability between biological replicates) was mostly higher for isolated trout hepatocytes than for carp hepatocytes.

**TABLE 2:** Mean *in vitro* intrinsic clearance (CL_in vitro, int,_ mL/h/10^6^ cells) ^1)^ of methoxychlor (MXC) and benzo[a]pyrene (BaP) in freshly isolated and cryopreserved common carp and rainbow trout hepatocytes.

Chemical	Cells	Carp				Trout			
		Mean	±SD	%CV	n	Mean	±SD	%CV	n
MXC	fresh	0.287^*^	0.051	17.8	6	0.250	0.151	60.5	5
	cryo	0.204	0.079	38.9	3	0.283	0.139	49.1	4
BaP	fresh	0.198^*^	0.034	17.4	6	0.251	0.074	29.3	4
	cryo	0.287	0.150	52.5	3	0.243	0.066	27.0	3

^1)^Values represent the mean ± standard deviation (SD) and coefficient of variation (%CV) over different *in vitro* runs for the respective substance and fish species. Values are normalized to 10^6^ cells, based on the nominal test concentration of 2 × 10^6^ cells/mL.

n = number of independent *in vitro* runs, each carried out in triplicates.

^*^Indicates significant differences (*p* = 0.015; Mann-Whitney Rank-sum) between MXC, and BaP.

### *In vivo* bioconcentration test

The results of the *in vivo* bioconcentration studies with rainbow trout and common carp are presented in detail in appendices (Supplementary Appendix A.2). In brief, common carp and rainbow trout were exposed simultaneously to 6.94 ± 1.66 and 11.45 ± 1.48 ng/L MXC, and 1.12 ± 0.34 and 1.62 ± 0.44 ng/L BaP (time-weighted average concentration ±SD, Supplementary Table A.2 6), respectively. Exposure concentrations below the solubility limit of the test items in water could be guaranteed due to the specific character of the column associated dosing system. The observed variabilities in the measured water concentrations (23.8 and 12.8% for MXC and 30.4 and 27.8% for BaP in carp and trout experiments, respectively), however, partly exhibited the allowed range of 20% according to OECD TG 305. This can be attributed to the very low test concentrations in combination with the long exposure period. With respect to water temperature (20.7 ± 0.08 for carp and 12.2 ± 0.07 for trout, average ± SD, Supplementary Figure A.2 1 and Supplementary Table A.2 1), dissolved oxygen concentration (77 ± 12.1% for carp and 91 ± 4.13% for trout, average ± SD, Supplementary Table A.2 2), and mortality (<10% at the end of the test) the test conditions met the OECD requirements for a valid test. At the end of the uptake period, mean lipid contents of 9.57 ± 1.48% and 9.08 ± 1.32% were measured for carp and trout, respectively (Supplementary Table A.2 4). Average body weights of carp and trout determined at the end of uptake were 44.6 ± 13.8 and 18.5 ± 7.10 g, respectively (Supplementary Table A.2 5).

*In vivo* k_B_ and BCF values measured for MXC and BaP in the two test species are summarized in Tables 3 and 4. Detailed data on the relevant bioconcentration parameters are contained in appendices (Supplementary Appendix A.2, Supplementary Table A.2 7, Supplementary Figure A.2 3, Supplementary Table A.2 8). Experimentally determined k_B_ values for carp compared to trout were 0.48 and 0.40 1/d for BaP as well as 0.21 and 0.10 1/d for MXC, respectively, with calculated uncertainty factors (95% probability confidences factors of *in vivo* k_B_ values, see Table 3) ranging from 2.2 to 3. Significant species differences of *in vivo* k_B_ values are thus not indicated for both chemicals. The experimentally determined BCF values differed significantly between the two test species: rainbow trout showed significantly higher BCF values than common carp in the case of MXC (2,686 compared to 1,188 L/kg), whereas the situation was opposite in the case of BaP: trout displayed significantly lower BCF values for BaP than did carp (77.7 compared to 140 L/kg). Measured BCF values differed significantly between MXC and BaP, with MXC showing much higher bioaccumulation than BaP in both species.

**TABLE 3:** *In vitro* − *in vivo* extrapolated whole body biotransformation rate constants (k_B_) of methoxychlor (MXC) and benzo[a]pyrene (BaP) vs *in vivo* derived k_B_ (k_B,__*in vivo*_ _estimate_) values.

Chemical	k_B_ carp [1/d]^1)^			k_B,__*in vivo*_ _estimate_ Carp [1/d]^2)^	k_B_ trout [1/d]^1)^			k_B,__*in vivo*_ _estimate_ Trout [1/d]^2)^	k_B,N_ - QSAR [1/d]^3)^
	Mean	±SD	%CV		Mean	±SD	%CV		
MXC	0.08^*^	0.01	17.1	0.21	0.06	0.03	58.1	0.10	0.034
BaP	0.03^*^	0.00	16.9	0.48	0.03	0.01	28.0	0.40	0.48

Parameterization of the Nichols et al. (2013) model was adapted according to the *in vivo* situations as described in the methods. The *f*_*u*_ values were calculated according to Nichols et al. (2013). Predictions based on a fixed *f*_*u*_ value of 1 are provided in appendices (Supplementary Appendix A.3, Supplementary Table A.3 3).

1) Values represent the mean ± standard deviation (SD) and coefficient of variation (%CV) of predicted k_B_, calculated for different in vitro runs with freshly isolated hepatocytes for the respective substance and fish species (common carp or rainbow trout). See Table 1 for number of in vitro runs (n) used for the calculations.

2) Values were derived from the experimentally determined whole body k_T_ data in common carp and rainbow trout using the k_B_ mass balance estimation model of Arnot et al. (2008). The method includes a calculated uncertainty factor for the k_B_ estimate corresponding to 95% probabilities of the true value being in this range. The uncertainty factors for BaP are 3 for both species meaning there is a 95% probability the expected in vivo k_B_ is within a factor of 3. For example, for BaP for trout the range is 0.13–1.2 1/d and for carp it is 0.16–1.4. The calculated uncertainty factors for k_B_ for MXC, were 2.3 and 2.2 for trout and carp estimates, respectively.

3) Values are geomeans of 5 k_B_-QSAR, predictions (Arnot et al., 2009; Brown et al., 2012; Papa et al., 2014; Mansouri et al., 2018).

* Indicates significant differences (p < 0.001; t-test) between MXC, and BaP.

**TABLE 4:** Comparison of predicted and measured bioconcentration factor (BCF) values of methoxychlor (MXC) and benzo[a]pyrene (BaP) in common carp and rainbow trout.

Chemical	Predicted BCF incorporating in vitro-derived k_B_ [L/kg]^1)^			Predicted BCF with k_B_ = 0 [L/kg]	Measured BCF [L/kg] ±SD
	Mean	±SD	%CV		
Common carp					
MXC	2,743^a^ ^,^ ^b^	291	10.6	7,633	1,188 ± 207^a^ ^,^ ^b^
BaP	5,014^a^ ^,^ ^b^	474	9.46	13,377	140 ± 17.5^a^ ^,^ ^b^
Rainbow trout					
MXC	4,486^a^ ^,^ ^b^	1,363	30.4	8,910	2,686 ± 1,260^a^ ^,^ ^b^
BaP	7,422^a^ ^,^ ^b^	1,406	18.9	25,807	77.7 ± 19.7^a^ ^,^ ^b^

1 Values represent the mean ± standard deviation (SD) of different in vitro runs for the respective substance and fish species.

BCF, predictions were performed based on the model of Nichols et al. (2013), with either k_B_ = 0 or k_B_ = values predicted from carp and trout hepatocyte *in vitro* clearance data (freshly isolated cells). The parametrization of the Nichols et al. (2013) model for carp was performed as described in Material and Methods.

a Indicates significant differences between MXC, and BaP (*p* < 0.001 and *p* = 0.016, *t*-test for predicted BCFs, in carp and trout, respectively; *p* < 0.001, Mann-Whitney Rank-sum for measured BCFs, in carp and trout).

b Indicates significant differences between common carp and rainbow trout (*p* = 0.004 and 0.010, Mann-Whitney Rank-sum for predicted BCSs, of MXC, and BaP, respectively; *p* < 0.001, *t*-test for measured BCFs, of MXC, and BaP).

### *In vitro-in vivo* extrapolation of k_B_ values and comparison to *in vivo*-derived K_B_ values

The CL_in vitro, int_ values obtained from the *in vitro* assays with trout and carp hepatocytes were incorporated into the prediction model of Nichols et al. (2013) to predict the *in vivo* k_B_ values of MXC and BaP. Mean k_B_ values predicted from the clearance rates of freshly isolated cells of both species are presented in Table 3. The values ranged from 0.03 to 0.08 1/d depending on the fish species and the chemical. The variability in the extrapolated k_B_ values was reduced compared to the %CV of CL_in vitro, int_ rates. Overall, predicted k_B_ values were lower than experimentally determined *in vivo* k_B_ values (Table 3), what probably relates to the fact that the latter values reflect whole body biotransformation rather than liver biotransformation only.

Similar to the measured CL_in vitro, int_ data, the extrapolated k_B_ values of MXC and BaP were not significantly different between carp and trout. This agrees with the findings from the *in vivo* tests: the k_B_ values of MXC and BaP derived from the *in vivo* experiment were not significantly different between carp and trout (Table 3).

When comparing the predicted k_B_ values for the two test compounds, the extrapolations based on data of the carp hepatocytes predicted a significantly higher biotransformation rate for MXC than for BaP. Such a compound difference was not predicted for rainbow trout.

*In vitro − in vivo* extrapolations of k_B_ values were also carried out for CL_in vitro, int_ data derived from experiments with cryopreserved hepatocytes. The estimated k_B_ values were not statistically different from those obtained with freshly isolated cells. The results are therefore provided in appendices (Supplementary Appendix A.3, Supplementary Table A.3 1).

Table 3 also compares the new IVIVE k_B_ values and empirical *in vivo* k_B_ estimates (obtained from the *in vivo* depuration experiments) with the geomean of 5 QSAR predictions (Arnot et al., 2009; Brown et al., 2012; Papa et al., 2014; Mansouri et al., 2018). For MXC, the *in vitro* derived k_B_ estimates (from liver tissue only) are within a factor of about 2-3 of both the *in vivo* and *in silico* k_B_ estimates. The *in vitro* values are slightly slower than the *in vivo* values and slightly faster than the QSAR predictions. For k_B_ BaP, the *in vitro* estimates (from liver tissue only) are slower than the *in vivo* and *in silico* estimates. Notably the BaP *in vitro* derived k_B_ estimates are about 1 order of magnitude slower than the *in vivo* and *in silico* k_B_ estimates, which are in very good agreement with each other. Collectively the *in vitro*, *in vivo* and *in silico* data demonstrate the utility of a weight-of-evidence approach for characterizing variability and uncertainty in k_B_ that can be considered when parameterizing bioaccumulation models.

### *In vitro-*based prediction of BCF values and comparison to *in vivo-*derived BCF values

The BCF values for carp and trout resulting from the model predictions are shown in Table 4. Assuming no biotransformation (k_B_ = 0), predicted BCF values for carp were 7,633 L/kg for MXC and 13,377 L/kg for BaP. In the case of trout, predicted BCF values with k_B_ = 0 ranged from 8,910 L/kg for MXC to 25,807 L/kg for BaP. Thus in both species BaP was predicted to have more bioaccumulation potential than MXC reflecting the higher K_ow_ of the former.

When the predicted k_B_ values were incorporated in the BCF calculations, the BCF values were, in all cases, reduced (2–3.5 fold). In contrast to the predicted k_B_ values, the predicted BCF values differed between the two fish species: for both chemicals, the BCF values of carp were significantly lower than those of trout (2,743 and 5,014 compared to 4,486 and 7,422 L/kg for MXC and BaP, respectively). This is in accordance with the findings from the *in vivo* test for MXC (lower BCF values for carp compared to trout) but not for BaP, where higher BCF values for carp than for trout were observed. Predicted BCF values differed significantly between MXC and BaP, with BaP showing higher bioaccumulation than MXC in both species. This is in contrast to the findings from the *in vivo* test, where MXC showed significantly higher bioaccumulation than BaP in both fish species.

The BCF predictions obtained for cryopreserved cells yielded comparable results to those for freshly isolated cells and can be found in appendices (Supplementary Appendix A.3 and Supplementary Table A.3 2).

## Discussion

The purpose of this study was to explore the inter-species applicability of the *in vitro* fish hepatocyte assay as a source of biotransformation rate data in alternative testing strategies for bioconcentration assessment in fish. More specifically, we investigated whether the well-established *in vitro* protocols for a biotransformation assay with hepatocytes of the cold water species, rainbow trout, can be transferred to the warm water species, common carp, and if the *in vitro* assays are able to predict *in vivo* species differences of BCF values.

### Technical transferability of the *in vitro* assay protocol

The technical transferability is not a trivial aspect since trout and carp differ substantially in their liver morphology. In contrast to salmonids, the non-capsule liver of carp is diffuse and intertwined with intestines, and there is not a single, large portal vein but many small venous connections between intestine and liver (Segner, 1998). It is therefore not possible to directly transfer the liver perfusion technique as it is routinely used for the isolation of hepatocytes from rainbow trout (Fay et al., 2015). As alternative to perfusion-based cell isolation, previous studies with carp hepatocytes relied on the *ex vivo* digestion of carp liver tissue with collagenase and trypsin (Cowan-Ellsberry et al., 2008; Dyer et al., 2008). However, the *in situ* digestion gives lower cell yields and is comparatively stressful for the cells, what may result in lower cell quality (Braunbeck and Segner, 2000). In the present study, we used a carp liver perfusion technique via the *Arteria coeliaca* according to Segner et al. (1993). This enabled us to isolate carp liver cells under comparable conditions as it is done with trout hepatocytes.

The cell yields obtained in the present study for carp liver (viable cells per g liver on average: 63.4 ± 13.2 × 10^6^) were comparable to the cell yields for rainbow trout liver in the present study (viable cells per g liver on average: 81.4 ± 31.2 ×10^6^) and to the values for trout liver of previous publications (Han et al., 2007; Mingoia et al., 2010; Fay et al., 2014a, 2017; Fay et al., 2014b). The carp hepatocyte yield of our study was also in close agreement to the cell yield for carp reported by Dyer et al. (Dyer et al., 2008, 2009) of 84 ± 21 × 10^6^ hepatocytes per Gram liver, and it corresponds to the data of Smeets et al. (1999) of 150–400 × 10^6^ cells per carp (our study: 402 × 10^6^ viable cells per fish after multiplication with the mean carp liver weight). The *in vitro* substrate depletion measurements worked well with the isolated carp hepatocytes. Substrate depletion followed first order kinetics in carp hepatocyte suspensions, as it did with trout liver cells, as evident from the R^2^-values of the log-linear decay curves of 0.85–0.99 for carp and 0.80–0.99 for trout.

A further indicator of the quality of *in vitro* hepatocyte preparations can be the intra- and inter-assay variability. Intra-assay variabilities of the carp hepatocyte assay were comparable to those of the trout hepatocyte assay (coefficients of variation (CV) between replicates of an *in vitro* run for MXC: 2–11% carp and 3–16% trout, and for BaP: 6–23% carp and 5–18% trout). In previous studies, for the rainbow trout hepatocyte assay up to 50% variability between cell preparations from different individual fish were reported (Fay et al., 2017). Depending on the test chemical under investigation, an inter-assay variability of up to 30% was determined for rainbow trout (Mingoia et al., 2010; Fay et al., 2014b). The variability of CL_in vitro, int_ across different laboratories was determined with up to 61% in a recent study using the trout hepatocyte assay (Fay et al., 2014b). Using freshly isolated hepatocytes of individual rainbow trout to measure *in vitro* metabolic rate values of difficult test compounds, Kropf et al. (2020) obtained inter-assay variabilities of 11–59%. In our study, measured total coefficients of variation (CV) of CL_in vitro, int_ ranged from 17.8 to 60.5% for MXC and from 17.4 to 52.5% for BaP. Thus, the inter-assay variabilities observed for the trout and carp hepatocyte assay in the present study were within the range of the results previously reported for trout.

Interestingly, a smaller variability of CL_in vitro, int_ rates between independent biological replicates was observed for hepatocyte suspensions from carp compared to trout, suggesting that inter-individual differences in the biotransformation potential may be less pronounced for carp in comparison to trout. This observation is consistent with the findings from a previous feeding study on common carp and rainbow trout (Bischof et al., 2016) which was performed with the same batches of fish as used for cell isolations in the present study. It was found that compared to trout, the concentration of MXC residues and the proportion of formed MXC metabolites in liver and fillet of carp varied less between different animals. More research is needed to evaluate whether the observed differences between carp and trout are the result of a different homogeneity of the particular fish strain or batch, or truly can be attributed to species-specific differences. If different fish species may be more or less subject to inter-individual differences in the metabolic potential, such inter-species differences could be an important criterion for the choice of fish species used for the hepatocyte assay.

A prerequisite for the commercial application and widespread use of primary fish hepatocytes for bioaccumulation assessment is the availability of a cryopreservation procedure that enables the storing and shipping of well-characterized batches of high quality hepatocytes. As demonstrated earlier, rainbow trout hepatocytes can be successfully cryopreserved without loss of biotransformation activity (Mingoia et al., 2010). The findings from the present study indicate that established protocols for trout hepatocyte cryopreservation can be successfully transferred to carp hepatocytes. Cell recoveries obtained with cryopreserved carp hepatocytes after thawing (percentage of viable cells in relation to the number of cells initially cryopreserved) were similar to those for trout and well in accordance to previously reported recoveries for trout of 22–37% (Fay et al., 2014a; Fay et al., 2014b). Also with respect to the cell viability, no significant differences were found between cryopreserved hepatocytes from carp and trout. Only towards the end of the *in vitro* incubation period, the biotransformation activity of cryopreserved and thawed carp hepatocytes slowed down. This was not the case with the cryopreserved and thawed trout liver cells.

In conclusion, the results of this study suggest that the standard protocols for the substrate depletion assay and the cryopreservation techniques as established for trout hepatocytes can be transferred to carp hepatocytes without the need for substantial modifications.

### Inter-species differences of biotransformation rates: Can they be predicted from *in vitro* assays*?*

Biotransformation rates vary between species (Schultz and Hayton, 1999; Han et al., 2007; Connors et al., 2013; Hutchinson et al., 2014). The question is whether *in vitro* preparations and the related *in vitro-in vivo* predictions reflect such species differences. Metabolic enzyme activities can differ significantly between mammalian and piscine *in vitro* systems (Nabb et al., 2006; Han et al., 2009). For fish, it has been shown that *in vitro* liver preparations are well able to unravel species differences in the patterns of the produced metabolites (Segner and Cravedi, 2001; Bischof et al., 2016). Also differences of biotransformation rates have been found between *in vitro* hepatic systems of different fish species (Fitzsimmons et al., 2007). Schultz and Hayton (1999) found over 25-fold variation of trifluralin metabolism in liver S9 preparations of rainbow trout, bluegill (*Lepomis macrochirus*), fathead minnow (*Pimephales*), lake sturgeon (*Acipenser fulvescens*), gizzard shad (*Dorosoma cepedianum*), channel catfish (*Ictalurus punctatus*) and largemouth bass (*Micropterus salmoides*). Roberts et al. (2011) investigated the *in vitro* metabolism of eleven individual polybrominated diphenyl ethers (PBDE) in three fish species and found that metabolite formation rates in carp liver microsomes were in general 10 to 100 times faster than those measured in trout and salmon. Strobel et al. (2015) reported significantly different *in vitro* biotransformation rates of BaP between Antarctic fish species and rainbow trout. Different *in vitro* biotransformation rates between rainbow trout and common carp liver microsomes were also determined for two surfactants in a study by Dyer et al. (2008).

While the available evidence suggests that *in vitro* liver preparations from different fish species metabolize identical chemicals at different rates, the question is whether these differences are predictive of the *in vivo* differences. A principal limitation in evaluating the predictivity of *in vitro* data is the availability of reliable *in vivo* data. For our test chemicals, MXC and BaP, we were not able to find BCFs of comparable quality between carp and trout. Therefore, we decided to perform an *in vivo* bioconcentration test according to OECD TG 305 for both test compounds and both test species. The results of the *in vivo* experiment revealed significant species differences of the BCF values: The measured *in vivo* BCF of MXC was significantly higher in trout than in carp, while the *in vivo* BCF of BaP showed no significant species difference. Interestingly, the species difference of the *in vivo* BCF values was not accompanied by a species difference of the *k*_*B*_ values derived from the *in vivo* experiment.

The *in vitro* assay with trout and carp hepatocytes displayed no significant differences of the intrinsic clearance rates for MXC and BaP. Likewise, the *k*_*B*_ values of BaP and MXC predicted from the results of the *in vitro* assays showed no significant species difference. However, the IVIVE predicted BCF values of MXC and BaP for rainbow trout and common carp differed significantly. These findings may point to a role of systemic toxicokinetic processes in determining the *in vivo* bioconcentration of chemicals. Therefore, the critical question with respect to the ability of *in vitro* assays to predict *in vivo* species differences of chemical bioaccumulation is probably not if the measured *in vitro* Cl_in vitro, int_ values differ between liver preparations of the species but if the overall extrapolation model is able to reflect the toxicokinetically relevant differences between the species. A handicap in answering this question is that the currently available IVIVE prediction models are parameterized for trout, but nor for carp. In our calculations, we tried to populate the Nichols model with carp-specific data, however, this was only partly possible, as the database of basic physiological parameters of carp is limited. This may explain why there was still a partial discrepancy between the measured and the predicted BCF values: while both measured and predicted data indicate a significantly lower bioconcentration of MXC in carp compared to trout, measured and predicted values differed for BaP: The IVIVE method predicted a significantly lower BCF of BaP in carp than in trout, whereas in the *in vivo* experiment carp show a significantly higher bioconcentration of BaP than trout. In this context, the findings of Laue et al. (2020) are of interest who showed that current IVIVE models tend to overpredict *in vivo* BCF values, and that this is not improved by changing *in vitro* assay conditions, by use of alternate fu values or by species matching, but seems to be an inherent problem of the existing extrapolation models. Thus, currently the ability of *in vitro* assays to reveal species differences in biotransformation rates appears to be related both to characteristics of the *in vitro* assays and the parametrization of the prediction models.

In conclusion, the findings from this study provide for the first time information on the ability of *in vitro* biotransformation rate assays to predict *in vivo* species differences of chemical bioaccumulation. It is evident that the results from this study having tested just two compounds in two species allows no conclusive statement, but the study findings highlight that the key challenge in this field is probably not so much the technical transfer of the *in vitro* methodology from rainbow trout to other fish species, but the requirement for improved species-specific parameterization of the IVIVE models.

## Data Availability

The original contributions presented in the study are included in the article/Supplementary Material, further inquiries can be directed to the corresponding author.
