# Supplementary material for: In vitro biotransformation assays using fish liver cells: Comparing rainbow trout and carp hepatocytes
Source: Front Toxicol. 2022 Sep 23;4:1021880. doi: 10.3389/ftox.2022.1021880 (PMC9538944; doi:10.3389/ftox.2022.1021880)
Supplement: Supplementary file 1 [file DataSheet1.pdf]

Supplementary Material

Appendices

A.1 Chemical analysis

Samples from the *in vitro* incubations containing MXC and methylene chloride extracts of water samples from the *in vivo* bioconcentration test were analyzed by gas chromatography-mass spectrometry (GC-MS). The GC-MS system consisted of a HP5890 series II GC coupled to a HP5972 mass sensitive detector (Hewlett-Packard). Separations were performed on 5% phenyl polysilphenylene-siloxane capillary columns (BPX-5, 30 m length x 0.25 mm i.d., 0.25 µm d_f, SGE). The carrier gas was helium at a constant flow of 1.2 mL/min. Oven temperature was programmed from 60°C to 280°C at a rate of 20°C/min with initial and final hold times of 3 and 5 min, respectively. Injections (2 µL) were performed with a HP7376 autosampler (Agilent, Hewlett-Packard) in splitless mode (1 min) at 280°C. The electron energy was set at 70 eV and the MS source temperature was set at 166°C. The MSD was operated in selected ion monitoring (SIM) mode at *m/z* 233 for the internal standard (MXC-d_6) and at *m/z* 227 and 229 for the analytes (MXC and ^{14}C-MXC), respectively, each with a dwell time of 100 msec. Nine-point calibration curves were acquired in a range of 15-300 ng/mL using 140 ng/mL MXC-d_6 as internal standard. The LOQ was determined at 13.7 ng/mL according to DIN32645.

Analysis of BaP in *in vitro* and water samples was carried out by liquid chromatography (LC)-fluorescence detection on a HPLC Summit system equipped with an Ultimate 3000 pump and a RF 2000 fluorescence detector (Dionex). Chromatography was performed on a Luna C18 100A column (Phenomenex, 250 mm × 4.6 mm, 5 µm particle size). The aqueous phase contained 100% deionized water, while the organic phase consisted of 100% ACN. Samples were eluted by running a linear gradient of 10-100% organic phase in 8 min and by a subsequent isocratic flow (100% organic phase) for 2 more min. The flow rate was (1 mL/min). BaP was detected using excitation/emission wavelengths of 290 nm and 430 nm.

Toluol extracts of fish samples from the *in vivo* bioconcentration studies were analyzed for MXC and BaP by GC-MS using a Varian Bruker 450-GC coupled to Varian Bruker 320-MS. Separations were performed on 1,4-bis(dimethylsiloxy)phenylene dimethyl polysiloxane capillary columns (Rxi®-5Sil MS, 20 m length x 0.18 mm i.d., 0.18 µm d_f, Restek). The carrier gas was helium at a constant flow of 1.0 mL/min. Oven temperature program started at 130°C for 1.5 min, increased by 25°C/min, and ended at 310°C for 6 min. Injections (1 µL) were performed with a CombiPal autosampler (CTC, equipped with a 10 µL Hamilton syringe) and a 1177 injector (Varian Bruker) in splitless mode (1.5 min) at 225°C. The mass spectrometer (MS) was operated in electrospray ionization (ESI) mode at an electron energy of 70 eV and a source temperature of 250°C.

For BaP, the MS was operated in SIM mode, detecting ions with *m/z* values 252.0 (BaP) and 264.1 (BaP-d_{12}). The dwell time was 150 msec.

For MXC, the MS was operated in MS/MS mode with the following transitions:

MXC: *m/z* 227.1 → 168.9 (collision energy 22V, quantifier)

MXC: *m/z* 227.1 → 211.9 (collision energy 13V, qualifier)

MXC-d_6: *m/z* 233.1 → 168.9 (collision energy 22V, quantifier)

MXC-d_6: *m/z* 233.1 → 215.0 (collision energy 13V, qualifier)

The dwell was 75 msec and as collision gas argon was used.
For BaP, eight-point calibration curves were acquired in a range of 0.2–20 ng/mL using 4 ng/mL BaP-d_{12} as internal standard. For MXC, ten-point calibration curves were acquired in a range of 0.5–300 ng/mL using 10 ng/mL MXC-d_6 as internal standard. Data were evaluated by means of Varian Workstation 6.9.3 software. Measured concentrations for sample dry weight were related to fresh weight using the measured dry weight – wet weight ratio of the sample.

A.2 *In vivo* bioconcentration test results

1 Water parameters

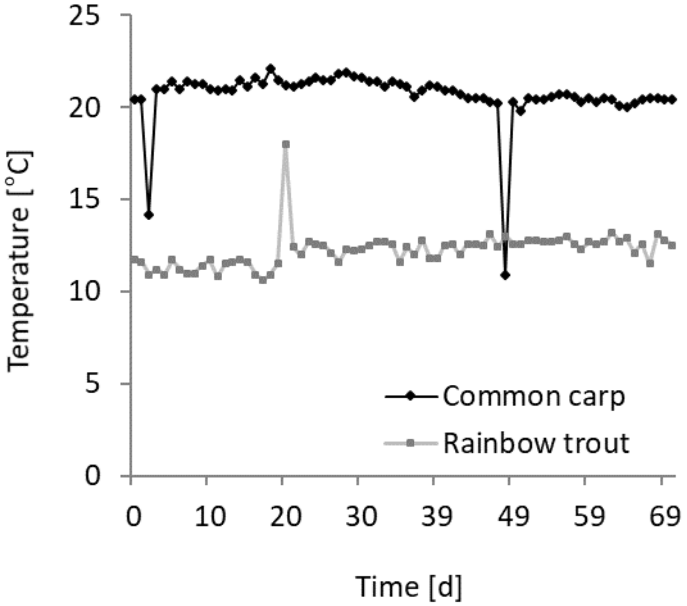

Supplementary Figure A.2-1. Measured temperatures in water of the test vessels during the *in vivo* fish bioconcentration studies.

Supplementary Table A.2-1: Mean temperature in water of the test vessels during the *in vivo* bioconcentration studies (n=72). SD= standard deviation; n= number of samples.

	Temperature [°C]	
	Common carp	Rainbow trout
Mean	20.7	12.2
Minimum value	10.9	10.6
Maximum value	22.1	18.0
SD	0.07	0.08

Supplementary Table A.2-2: Mean oxygen saturation and pH values in water of the test vessels during the *in vivo* fish bioconcentration studies (n=72).

	**pH**		**Oxygen [mg/L]**		**0_2-Saturation [%]**	
	Common carp	Rainbow trout	Common carp	Rainbow trout	Common carp	Rainbow trout
Mean (oxygen) or median (pH)	7.65	7.92	6.6	7.9	77	91
Minimum value	7.39	7.74	4.7	6.6	56	81
Maximum value	8.00	8.58	8.4	9.1	97	101
SD	0.15	0.15	1.10	0.58	12.1	4.13

SD= standard deviation; n= number of samples.

Supplementary Table A.2-3: Weekly measured values of non-purgeable organic carbon (NPOC) in water of the test vessels during the *in vivo* bioconcentration studies (n=13)**.**

	**NPOC [mg/L]**	
	Common carp	Rainbow trout
Mean	0.91	1.04
Minimum value	1.43	1.98
Maximum value	1.43	1.98
SD	0.32	0.35

SD= standard deviation; n= number of samples.

2 Growth performance of fish

Supplementary Table A.2-4: Lipid content of fish during the *in vivo* bioconcentration study *.

	Common carp			Rainbow trout		
day	Lipid [%]	Mean lipid [%]	±SD	Lipid [%]	Mean lipid [%]	±SD
0	8.42	9.30	0.76	7.08	6.05	1.19
0	9.66			6.68		
0	9.80			6.08		
0	/			4.37		
35	10.8	9.57	1.48	8.41	9.08	1.32
35	9.99			8.23		
35	7.92			10.6		
70	11.8	12.5	2.31	11.5	10.7	1.17
70	15.1			9.86		
70	10.6			/		

* Fish used for lipid analysis were sampled in addition to the fish sampled for chemical analysis and were used for lipid analysis only.

/= no fish sampled; SD= standard deviation.

Supplementary Table A.2-5: Body weight of fish at the beginning and end of uptake (days 0 and 35) and depuration period (days 35 and 70) of the *in vivo* bioconcentration study ***.**

* Individual body weights were determined for all sampling time points. The natural logarithm transformed data is presented in Figure A.2-2; SD= standard deviation.

	Common carp			Rainbow trout		
day	Weight [g]	Mean weight [g]	±SD	Weight [g]	Mean weight [g]	±SD
0	33.2			6.80		
0	40.9	41.9	6.44	10.1	8.68	1.60
0	47.6			9.90		
0	45.8			7.90		
35	25.5			23.2		
35	47.0	44.6	13.8	23.1	18.5	7.10
35	58.6			19.4		
35	47.4			8.14		
70	87.4			66.5		
70	83.7	74.5	17.2	38.7	48.6	15.5
70	77.3			6.80		
70	49.3			10.1		

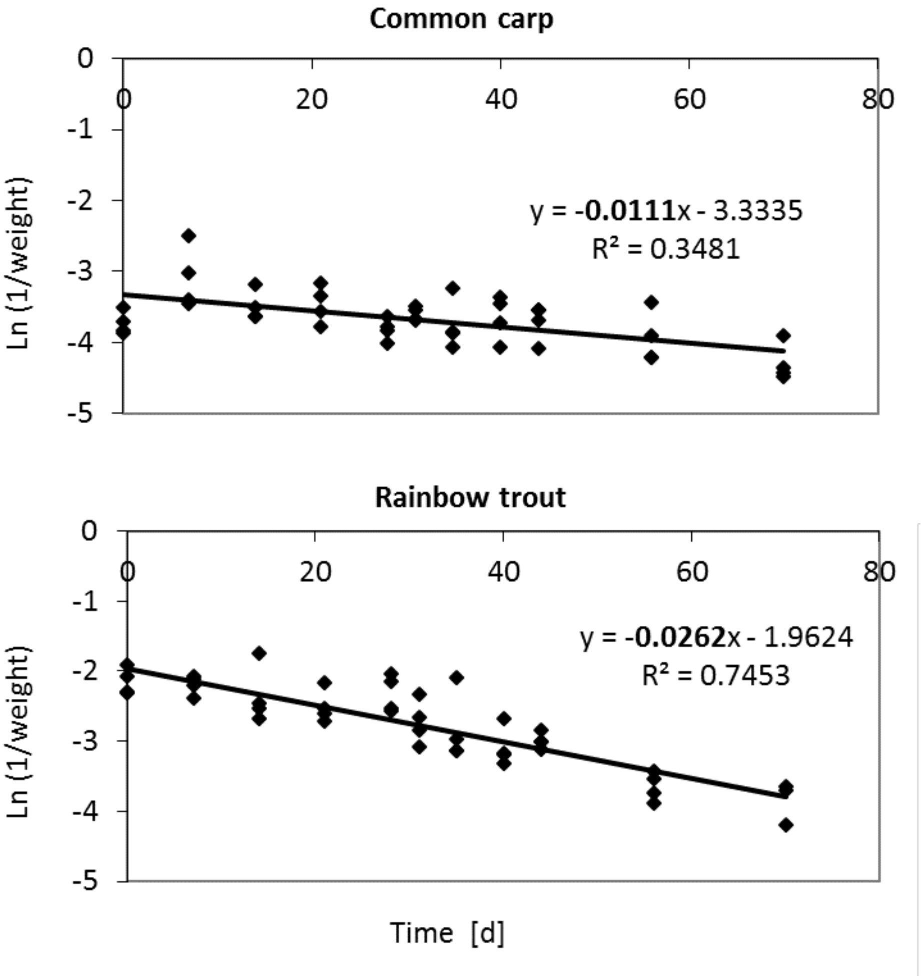

Supplementary Figure A.2-2. Calculated overall (uptake + depuration phase) growth rate constant (k_G expressed as 1/d), as determined from the slope of the linear regression curve of natural logarithms of individual weight data plotted against time. Overall growth rate constants were used for the growth correction of the calculated depuration rate constants k_T.

3 Measured chemical concentrations in water and fish

Supplementary Table A.2-6: Methoxychlor (MXC) and benzo[a]pyrene (BaP) concentrations measured in water (C_w) of the test vessels during the *in vivo* bioconcentration studies.

Time of exposure [d]	BaP concentration [ng/L]		MXC concentration [ng/L]	
	Carp	Trout	Carp	Trout
0	4.85*	3.72*	18.02*	20.63*
1	0.21	2.65	7.90	12.90
2	1.39	2.27	10.13	13.25
5	1.42	1.13	8.80	14.24
6	1.09	1.07	7.80	12.93
7	1.05	1.21	6.20	11.36
8	1.05	1.46	7.45	13.18
9	1.27	1.55	5.51	11.23
12	1.35	1.88	6.28	11.32
14	0.86	1.05	5.47	10.60
16	1.16	2.08	9.11	12.27
19	1.28	1.42	5.63	9.30
21	1.32	1.68	4.88	10.71
23	0.90	1.29	6.40	12.45
26	1.57	1.86	9.50	12.50
28	0.62	1.32	4.88	9.33
30	0.77	1.24	4.98	9.85
33	1.12	1.90	7.32	10.52
35	1.44	1.75	7.23	9.90
Overall mean	1.10	1.60	6.97	11.55
TWA	1.12	1.62	6.94	11.45
Min	0.21	1.05	4.88	9.30
Max	1.57	2.65	10.13	14.24
SD	0.34	0.44	1.66	1.48
%CV	30.43	27.78	23.75	12.81

TWA= Time-weighted average; SD= Standard deviation; %CV= Percent coefficient of variation.

* Values identified as outliers by the Grubb's test at 95% confidence level (SQS 2010 V1.45) and therefore excluded from further calculations.

Supplementary Table A.2-7: Methoxychlor (MXC) and benzo[a]pyrene (BaP) concentrations measured in fish (C_f) during the in vivo bioconcentration studies.

	MXC						BaP					
day	Common carp			Rainbow trout			Common carp			Rainbow trout		
	C_f data [µg/kg]	Mean C_f [µg/kg]	±SD	C_f data [µg/kg]	Mean C_f [µg/kg]	±SD	C_f data* [µg/kg]	Mean C_f [µg/kg]	±SD	C_f data* [µg/kg]	Mean C_f [µg/kg]	±SD
0	0.000			0.000			0.000			0.000		
0	0.023	0.006	0.011	0.000	0.000	0.000	0.000	0.001	0.002	0.002	0.001	0.001
0	0.000			0.000			0.000			0.000		
0	0.000			0.000			0.005			0.001		
7	12.8			25.9			0.204			0.060		
7	11.7	12.2	1.34	30.0	23.0	5.98	0.175	0.216	0.043	0.095	0.107	0.040
7	10.7			18.7			0.207			0.117		
7	13.8			17.4			0.277			0.157		
14	11.4			27.1			0.168			0.079		
14	9.30	9.88	1.10	18.1	21.1	6.32	0.141	0.172	0.026	0.073	0.070	0.008
14	10.0			25.4			0.205			0.070		
14	8.83			13.7			0.176			0.060		
21	9.16			28.0			0.136			0.111		
21	9.96			23.9			0.179			0.107		
		8.56	1.30		21.5	5.81		0.154	0.018		0.141	0.039
21	6.95			14.5			0.155			0.160		
21	8.16			19.4			0.147			0.186		
28	7.02			53.2			0.130			0.126		
28	5.90	6.94	0.80	50.1	41.9	11.6	0.133	0.148	0.019	0.140	0.121	0.022
28	7.85			34.5			0.159			0.128		
28	7.00			29.6			0.169			0.088		
35	8.35			50.9			0.149			0.084		
35	7.32	7.59	0.50	49.5	46.4	16.2	0.143	0.153	0.008	0.086	0.116	0.036
35	7.30			23.5			0.161			0.147		
35	7.40			61.7			0.159			0.146		

	**MXC**						**BaP**					
day	Common carp			Rainbow trout			Common carp			Rainbow trout		
	C_f data [µg/kg]	Mean C_f [µg/kg]	±SD	C_f data [µg/kg]	Mean C_f [µg/kg]	±SD	C_f data* [µg/kg]	Mean C_f [µg/kg]	±SD	C_f data* [µg/kg]	Mean C_f [µg/kg]	±SD
40	3.02			/			0.017			0.008		
40	2.71	2.40	0.57	9.62	13.8	3.61	0.019	0.013	0.007	0.013	0.011	0.004
40	1.73			15.9			0.007			0.007		
40	2.16			15.8			0.007			0.016		
44	0.819			2.19			0.003			0.004		
44	0.719	0.757	0.177	7.53	6.06	2.70	0.004	0.002	0.002	0.002	0.003	0.001
44	0.956			8.23			0.000			0.002		
44	0.535			6.28			0.000			0.002		
56	-			5.42 **			-			-		
56	-	-	-	2.06 [a]	1.75 [a]	0.68 [a]	-	-	-	-	-	-
56	-			2.23 [a]			-			-		
56	-			0.969 [a]			-			-		
70	-			2.46 [a]			-			-		
70	-	-	-	0.320 [a]	1.01 [a]	1.26 [a]	-	-	-	-	-	-
70	-			0.237 [a]			-			-		
70	-			/			/			-		

* Shown concentrations are corrected for mean background BaP levels measured in samples of day 0.

** Value identified as outlier by the Grubb's test at 95% confidence level (SQS 2010 V1.45) and therefore excluded from further calculations.

"-" Measured concentrations were below the LOQ (0.258 µg/kg for MXC and 0.0129 µg/kg for BaP before background correction) and were therefore excluded from further calculation.

"/" Not determined, lost sample.

[a] Day 70 values excluded from k_T calculation and day 56-70 values excluded from k_T-high calculation in order to improve fit (see Figure A.2-3).

4 Determination of *in vivo* bioconcentration parameters

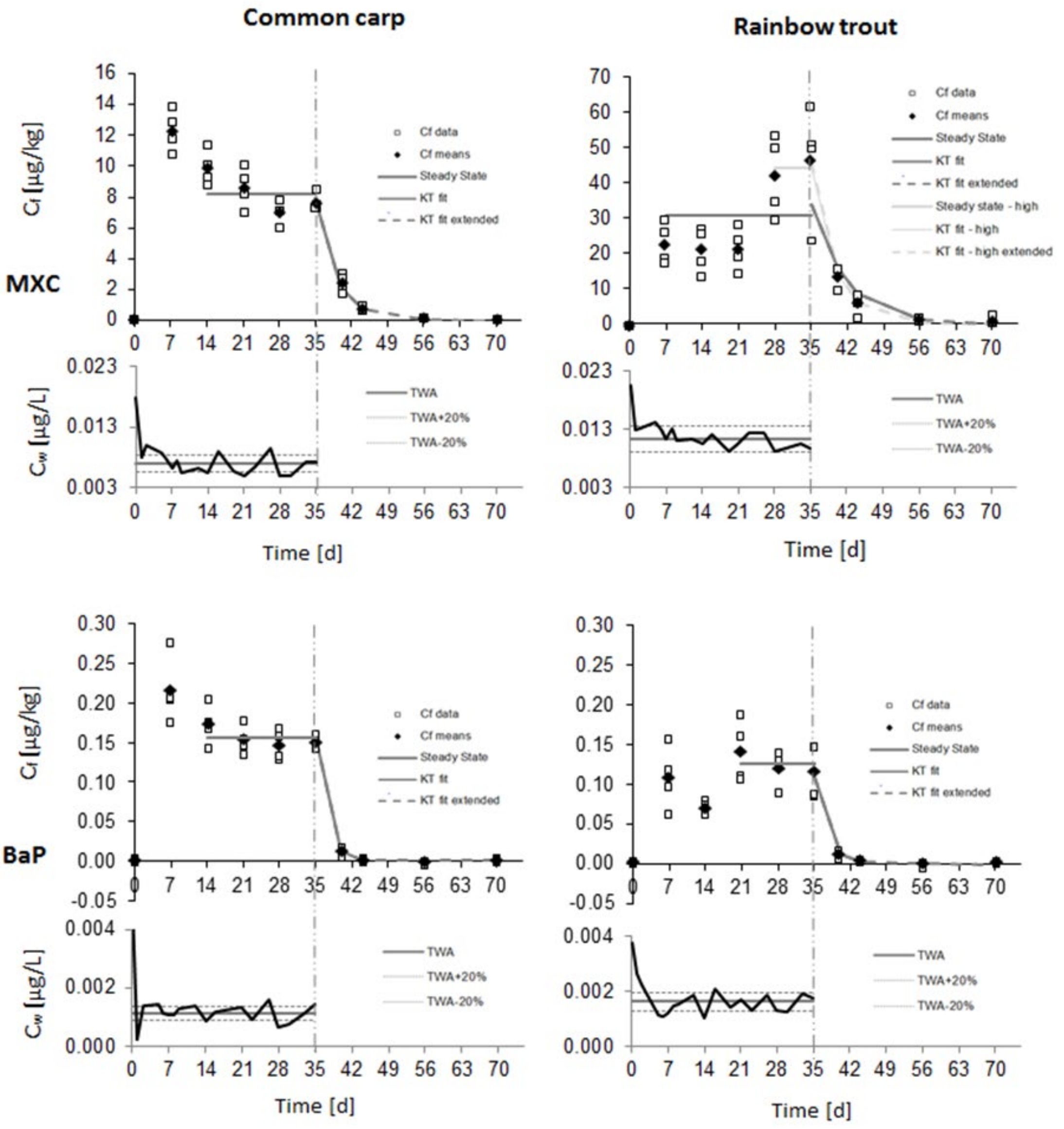

Supplementary Figure A.2-3: Determination of steady-state BCF (BCF_{SS}) and depuration rate constant (k_T). Measured concentrations of methoxychlor (MXC) and benzo[a]pyrene (BaP) in fish (C_f) and in water (C_w) during the uptake (day 0-35) and depuration (day 35-70) phase of the *in vivo* bioconcentration studies. Steady-state concentrations in fish and time-weighted average (TWA) concentrations in water used for the calculation of BCF_{SS} are indicated with a parallel grey line. The lower and upper limit of the allowed range of variability of test item concentrations in water from the TWA is indicated by a dotted grey line. The grey line in the depuration phase shows the fitted depuration curve as determined from the intercept and slope (k_T) of the linear regression of ln-transformed C_f data versus time. The dotted grey line shows the curve progression of the first order kinetic model outside the time points used for fitting. Visual inspection of the modeled curve plotted against the measured sample point data confirms a good fit. The increase in measured concentrations of MXC in rainbow trout after day 21 might be attributed to some biological artifact such as a break-down in metabolism. The data was therefore used to calculate a second, higher steady-state of day 28 and 35 as indicated by the lighter grey color.

Supplementary Table A.2-8: Experimentally determined and modeled bioconcentration parameters for the *in vivo* bioconcentration studies.

	MXC					
Parameter	Common carp		Rainbow trout		Rainbow trout - high BCF_{SS}	
$C_{f,SS}$ [µg/kg] ±%SD	8.24	15.5	30.76	40.09	44.1	7.26
TWA C_w [ng/L] ±SD	6.94	1.65	11.45	1.47	11.45	1.47
BCF_{SS} [L/kg] ±SD	1188	207	2686	1260	3854	1158
BCF_{SSL} [L/kg] ±SD	621	108	1479	694	2122	638
k_T [1/d] (R^2)	0.255	(0.996)	0.150	(0.948)	0.227	(0.998)
K_G [1/d]	0.0111	n.d.	0.0262	n.d.	0.026	n.d.
k_{TG} [1/d]	0.244	n.d.	0.124	n.d.	0.201	n.d.
$k_{1,modeled}$ [L/kg/d]	348	n.d.	251	n.d.	251	n.d.
$K_{B, modeled}$ [1/d] + CF	0.21	2.2	0.10	2.3	0.18	2.2

	BaP			
Parameter	Common carp		Rainbow trout	
$C_{f,SS}$ [µg/kg] ±%SD	0.157	6.91	0.126	10.63
TWA C_w [ng/L] ±SD	1.12	0.34	1.62	0.45
BCF_{SS} [L/kg] ±SD	140	17.5	77.7	19.7
BCF_{SSL} [L/kg] ±SD	73	9.13	42.8	10.8
k_T [1/d] (R^2)	0.493	(1.00)	0.427	(0.996)
K_G [1/d]	0.0111	n.d.	0.0262	n.d.
k_{TG} [1/d]	0.482	n.d.	0.401	n.d.
$k_{1,modeled}$ [L/kg/d]	348	n.d.	251	n.d.
$K_{B\ modeled}$ [1/d] + CF	0.48	3.00	0.40	3.00

n.d. = not determined; $C_{f,SS}$= concentration in fish at steady-state.

TWA C_w= time-weighted average concentration in water during the uptake phase.

BCF_{SS}= steady-state bioconcentration factor; BCF_{SSL}= lipid-normalized steady-state bioconcentration factor (5% lipid content).

k_T= depuration rate constant; k_G= overall growth rate constant; k_{TG}= growth corrected depuration rate constant; $k_{1,modeled}$= modeled uptake rate constant.

$K_{B\ modeled}$= whole body biotransformation rate constant; CF= confidence factor.

A.3 *In vitro – in vivo* extrapolation of k_B and BCF: data from cryopreserved hepatocytes

Supplementary Table A.3-1: *In vitro – in vivo* extrapolated whole body biotransformation rate constants (k_B [1/d] [1)] of methoxychlor (MXC) and benzo[a]pyrene (BaP) in common carp and rainbow trout calculated based on *in vitro* data from cryopreserved cells.

	Common carp			Rainbow trout		
Chemical	Mean	±SD	%CV	Mean	±SD	%CV
MXC	0.05	0.02	37.8	0.07	0.03	48.2
BaP	0.04	0.02	51.4	0.03	0.01	26.1

[1)] Values represent the mean ± standard deviation (SD) and coefficient of variation (%CV) of k_B calculated for different *in vitro* runs with cryopreserved cells for the respective substance and fish species.

For the *in vitro-in vivo* prediction, the model of Nichols et al. (2013) was used. Parametrization of the model was adapted for carp as described in the methods.

Supplementary Table A.3-2: Predicted bioconcentration factor (BCF) values [L/kg] 1) of methoxychlor (MXC) and benzo[a]pyrene (BaP) in common carp and rainbow trout after incorporating the in vitro-based kB values derived from cryopreserved cells.

	Common carp			Rainbow trout		
Chemical	Mean	±SD	%CV	Mean	±SD	%CV
MXC	3420	669	19.5	4200*	1555	37.0
BaP	4336	1724	39.8	7546*	1440	19.1

[1)] Values represent the mean ± standard deviation (SD) of different *in vitro* runs for the respective substance and fish species.

For the *in vitro-in vivo* prediction, the model of Nichols et al. (2013) was used. Parametrization of the model was adapted for carp as described in the methods.

[*] Indicates significant differences between MXC and BaP (P=0.034, t-test).

Supplementary Table A.3-3: *In vitro − in vivo* extrapolated whole body biotransformation rate constants (k_B) [1/d] [1)] of methoxychlor (MXC) and benzo[a]pyrene (BaP) in common carp and rainbow trout calculated based on *in vitro* data from freshly isolated cells and under the assumption that f_u=1 instead of f_u=modeled.

	**Common carp**			**Rainbow trout**		
Chemical	Mean	±SD	%CV	Mean	±SD	%CV
MXC	1.49[a,b]	0.09	5.95	0.87[a,b]	0.18	20.4
BaP	0.73[a,b]	0.05	6.79	0.52[a,b]	0.04	7.73

[1)] Values represent the mean ± standard deviation (SD) and coefficient of variation (%CV) of k_B calculated for different *in vitro* runs with freshly isolated cells for the respective substance and fish species.

For the *in vitro-in vivo* prediction, the model of Nichols et al. (2013) was used. Parametrization of the model was adapted for carp as described in the methods.

[a] Indicates significant differences between MXC and BaP ($P<0.001$, t-test for carp and $P=0.016$, Mann-Whitney Rank-sum for trout).

[b] Indicates significant differences between common carp and rainbow trout ($P=0.004$, Mann-Whitney Rank-sum for MXC and $P<0.001$, t-test for BaP).

Supplementary Table A.3-4: Predicted bioconcentration factor (BCF) values [L/kg] [1)] of methoxychlor (MXC) and benzo[a]pyrene (BaP) in common carp and rainbow trout after incorporating the k_B estimate calculated based on *in vitro* data from freshly isolated cells and under the assumption that f_u=1 instead of f_u=modeled.

	**Common carp**			**Rainbow trout**		
Chemical	Mean	±SD	%CV	Mean	±SD	%CV
MXC	209[a,b]	11.9	5.70	507[b]	106	20.8
BaP	274[a,b]	17.0	6.22	543 [b]	40.4	7.44

[1)] Values represent the mean ± standard deviation (SD) of different *in vitro* runs for the respective substance and fish species.

For the *in vitro-in vivo* prediction, the model of Nichols et al. (2013) was used. Parametrization of the model was adapted for carp as described in the methods.

[a] Indicates significant differences between MXC and BaP ($P=0.001$, t-test).

[b] Indicates significant differences between common carp and rainbow trout ($P=0.004$ and 0.010, Mann-Whitney Rank-sum for MXC and BaP, respectively).
